# Supplementary material for: Integration of a clinical pharmacist workforce into newly forming primary care networks: a qualitatively driven, complex systems analysis
Source: BMJ Open. 2022 Nov 3;12(11):e066025. doi: 10.1136/bmjopen-2022-066025 (PMC9639102; doi:10.1136/bmjopen-2022-066025)
Supplement: Supplementary data [file bmjopen-2022-066025supp001.pdf]

Topic guide

- Details of PCN role – job title, when took up post, overview of current roles, brief career history
- PCN policy – views and experiences of PCN policy, relationship with colleagues during transition, autonomy to shape PCN direction, PCN level of maturity, type of operational model, pros and cons of operational model
- New clinical pharmacy workforce – potential of new clinical pharmacy workforce, suitability of national training, personal role in the integration of new clinical pharmacists, local arrangements for integrating, managing and supervising clinical pharmacists
- Medication reviews – personal approach to medication reviews, alcohol within medication reviews, understanding of the new Structured Medication Review (SMR), local implementation of SMRs
- Research – importance of research to PCN leadership, willingness to promote involvement of new clinical pharmacists in a trial of person-centred alcohol discussions in SMRs
