# Supplementary material for: Integration of a clinical pharmacist workforce into newly forming primary care networks: a qualitatively driven, complex systems analysis
Source: BMJ Open. 2022 Nov 3;12(11):e066025. doi: 10.1136/bmjopen-2022-066025 (PMC9639102; doi:10.1136/bmjopen-2022-066025)
Supplement: Author's manuscript [file bmjopen-2022-066025.draft_revisions.pdf]

BMJ Open is committed to open peer review. As part of this commitment we make the peer review history of every article we publish publicly available.

When an article is published we post the peer reviewers' comments and the authors' responses online. We also post the versions of the paper that were used during peer review. These are the versions that the peer review comments apply to.

The versions of the paper that follow are the versions that were submitted during the peer review process. They are not the versions of record or the final published versions. They should not be cited or distributed as the published version of this manuscript.

BMJ Open is an open access journal and the full, final, typeset and author-corrected version of record of the manuscript is available on our site with no access controls, subscription charges or pay-per-view fees (http://bmjopen.bmj.com).

If you have any questions on BMJ Open's open peer review process please email info.bmjopen@bmj.com

BMJ Open

The integration of a clinical pharmacist workforce into newly forming Primary Care Networks: a qualitatively-driven, complex systems analysis

Journal:	*BMJ Open*
Manuscript ID	bmjopen-2022-066025
Article Type:	Original research
Date Submitted by the Author:	27-Jun-2022

Complete List of Authors:	Mills, Thomas; University of York; London South Bank University<br>Madden, Mary; University of York,<br>Stewart, Duncan; London Metropolitan University,<br>Gough, Brendan; Leeds Beckett University<br>McCambridge, Jim; University of York

Keywords:	Health policy < HEALTH SERVICES ADMINISTRATION & MANAGEMENT, PRIMARY CARE, QUALITATIVE RESEARCH, Organisation of health services < HEALTH SERVICES ADMINISTRATION & MANAGEMENT, Change management < HEALTH SERVICES ADMINISTRATION & MANAGEMENT, International health services < HEALTH SERVICES ADMINISTRATION & MANAGEMENT

SCHOLARONE™
Manuscripts

I, the Submitting Author has the right to grant and does grant on behalf of all authors of the Work (as defined in the below author licence), an exclusive licence and/or a non-exclusive licence for contributions from authors who are: i) UK Crown employees; ii) where BMJ has agreed a CC-BY licence shall apply, and/or iii) in accordance with the terms applicable for US Federal Government officers or employees acting as part of their official duties; on a worldwide, perpetual, irrevocable, royalty-free basis to BMJ Publishing Group Ltd ("BMJ") its licensees and where the relevant Journal is co-owned by BMJ to the co-owners of the Journal, to publish the Work in this journal and any other BMJ products and to exploit all rights, as set out in our licence.

The Submitting Author accepts and understands that any supply made under these terms is made by BMJ to the Submitting Author unless you are acting as an employee on behalf of your employer or a postgraduate student of an affiliated institution which is paying any applicable article publishing charge ("APC") for Open Access articles. Where the Submitting Author wishes to make the Work available on an Open Access basis (and intends to pay the relevant APC), the terms of reuse of such Open Access shall be governed by a Creative Commons licence – details of these licences and which Creative Commons licence will apply to this Work are set out in our licence referred to above.

Other than as permitted in any relevant BMJ Author's Self Archiving Policies, I confirm this Work has not been accepted for publication elsewhere, is not being considered for publication elsewhere and does not duplicate material already published. I confirm all authors consent to publication of this Work and authorise the granting of this licence.

For peer review only

The integration of a clinical pharmacist workforce into newly forming Primary Care Networks: a qualitatively-driven, complex systems analysis

Title page

Author list:

Dr Thomas Mills, University of York, York, YO10 5DD

Dr Mary Madden (Associate Professor), University of York, York, YO10 5DD

Professor Duncan Stewart, London Metropolitan University, 166-220 Holloway Rd, London N7 8DB

Professor Brendan Gough, Leeds Beckett University, Leeds LS1 3HE

Professor Jim McCambridge, University of York, York, YO10 5DD

Corresponding author details:

Dr Thomas Mills

University of York, York, YO10 5DD

Email: millst3@lsbu.ac.uk

TEL: 07806497970

Fax: +44 (0)1904 32 3433

Key terms: Primary Health Care, General Practice, Pharmacy, Qualitative Research, Complex Systems Modelling

Word count: 4784

For peer review only

Abstract

Objective

The introduction of a new clinical pharmacist workforce via Primary Care Networks (PCNs) is a recent national policy development in the National Health Service (NHS) in England. This study elicits the perspectives of people with responsibility for local implementation of this national policy package. Attention to local delivery is necessary to understand the contextual factors shaping the integration of the new clinical pharmacy workforce, and thus can be expected to influence future role development.

Design

A qualitative, interview study

Setting and participants

PCN Clinical Directors and senior pharmacists across 17 PCNs in England (n=28)

Analysis

Interviews were transcribed, coded and organised using the framework method. Thematic analyses and complex systems modelling were then undertaken iteratively to develop the themes.

Results

Findings were organised into two overarching themes: 1) Local organisational innovations of a national policy under conditions of uncertainty; 2) Local multi-professional decision-making on clinical pharmacy workforce integration and initial task assignment. Although a phased implementation of the PCN package was planned, the findings suggest that processes of PCN formation and clinical pharmacist workforce integration were closely intertwined, with underpinning decisions taking place under conditions of considerable uncertainty and workforce pressures.

Conclusions

National policy decisions that required GPs to form PCNs at the same time as they integrated a new workforce risked undermining the potential of both PCNs and the new workforce. PCNs require time and support to fully form and integrate clinical pharmacists if successful role development is to occur. Efforts to incentivise delivery of PCN pharmacy services in future must be responsive to local capacity.

For peer review only

Strengths and Limitations

- The present study represents a rigorous and timely qualitative investigation of the early integration of a new clinical pharmacist workforce into primary care that is anticipated to take on a significant, patient-facing role in future.
- The sampling approach allowed the research to capture insights on the new clinical pharmacy workforce across 17 diverse PCNs in England.
- While the diversity of PCNs provided broad brush insight, a case study approach that focused on a smaller number of PCNs more in depth could provide deeper lessons.
- The principal study focus on the clinical pharmacist workforce means that the study does not explore other developments that are being implemented as part of the PCN policy package.
- The study focus on senior staff (PCN clinical directors and senior pharmacists) could also be complemented, in future, by the qualitative study of the perspectives of the newly integrated workforce itself.

For peer review only

Introduction

Reform efforts globally have sought to develop the primary care sector with the underpinning idea that the sector is vital to contemporary healthcare challenges(1). In the UK, a range of institutional reforms have, over the past four decades, been enacted to transform the traditional set-up of small private business partnerships run by General Practitioners (GPs). GPs have traditionally been contracted by the NHS to provide a range of services to a local population and serve as “gate keepers” to hospital-based specialist services. Reforms to this set-up have included attempts to enhance the role of GPs in healthcare commissioning and new models of service delivery that have transferred services out of hospitals into primary care(2, 3, 4, 5). There have also been efforts to address shortfalls in the numbers of GPs and meet new healthcare challenges, including facing the increasing demand and complexity of patient needs, through the integration and development of new primary care roles(6, 7). This reflects a broader task shifting to “mid-level” professionals who are educated and trained to a level where they can undertake some of the activities of GPs and specialists within a prescribed scope of practice(8, 9). In primary care, some GP work tasks and responsibilities have shifted to nursing staff(5) and, more recently, clinical pharmacists(7, 10). Such developments occur within ongoing contexts of reorganisations, funding constraints and other pressures(10).

The NHS Long Term Plan, published in January 2019, featured a new funding settlement for primary care (with projected funding to be £4.5 billion higher in 2023/24 than in 2019/20) and plans for new staff roles and services(11). In order to qualify for new funding, GPs were to collaborate with neighbouring practices to form Primary Care Networks (PCNs)(11, 12). The aim was to expand the primary care workforce and enhance patient care through new and improved services, based on aspects of Canadian(13) and New Zealand(14) primary care systems. A new national contract, the Network Contract Directed Enhanced Services (known colloquially as ‘the DES’), was introduced on the 1st of July 2019. This facilitated access to funds for new staff roles via the Additional Roles Reimbursement Scheme (ARRS). 1,250 PCNs were formed across England in the first year of the policy, each serving around 30-50,000 patients(12). Qualitative research undertaken during the PCN policy’s formation stage indicated potential for future, stakeholder disagreement, due to the presence of multiple, contrasting objectives(16); it was unclear, at the start of the current research, if such disagreement had complicated implementation.

This paper presents a qualitative investigation of the early integration of a new clinical pharmacy workforce into newly forming PCNs. Clinical pharmacists are a key example of a “mid-level” professional group(8, 9). Historically, pharmacists in the UK have mainly operated in secondary care hospitals or in community pharmacy, providing prescription dispensing services and, more recently, over-the-counter advice(15). Numbers of pharmacists in GP practices increased during the 1990s and 2000s but the absence of a national workforce strategy resulted in significant diversity, in terms of numbers and roles, across GP practices(15). A key long-term aim of PCN policy was to implement nationally a patient-facing clinical pharmacy role, following an initial pilot in 2016(6): PCNs were permitted to employ a single clinical pharmacist in their first year of formation (2019), with this number expected to rise to five clinical pharmacists per PCN

For peer review only - http://bmjopen.bmj.com/site/about/guidelines.xhtml

by 2024(12). The research aim was to investigate how the PCN policy package was being implemented locally and to develop understanding of the contextual factors that were enabling or constraining the integration of the new clinical pharmacy workforce.

Methods

A range of exploratory studies were previously undertaken to assess the potential of the pharmacist role with a view to incorporating attention to alcohol within medicine reviews(17, 18, 19). These studies featured within a research programme that started in community pharmacy and then moved into general practice as the new clinical pharmacist workforce was recruited there. The present study included interviews with senior staff with responsibility for planning and integrating the clinical pharmacy workforce into newly forming PCNs. The interviews took place between March 2020, a year after PCNs were established, through to September 2021, providing a snapshot of this aspect of the national policy package. Three sets of interviews were undertaken, creating three sub-study datasets:

a) Twelve semi-structured interviews with seven senior PCN staff (three GP Clinical Directors and four senior PCN pharmacists), between March 2020 and September 2021, in six PCNs based in the Yorkshire and Humber and North-East regions of England (i.e., two interviews one year apart to explore developments over time, although one participant changed role and declined the second interview). Quotation codes: CD (Clinical Director) and SP (Senior Pharmacist); the addition of b indicates second interview.

b) 10 one-off semi-structured interviews between March 2021 and August 2021 with 10 pharmacists in 10 PCNs who were already working in primary care prior to the formation of PCNs and were established in a senior role (either in a GP practice or a PCN) during the transition. Quotation codes: SPX.

c) Six semi-structured interviews conducted between September 2020 and June 2021 with three newly appointed senior pharmacists across 4 PCNs (i.e., two interviews one year apart to explore developments over time; one interviewee switched to a different PCN during the study). Quotation codes: SPY; the addition of b indicates second interview.

The sampling strategy for sub-study a) was pragmatic and opportunistic: we initially sought diversity of PCNs, in terms of operational models and patient populations, but this was not possible to ensure because PCNs were still forming. Changing PCN characteristics were therefore explored through the interviews. Sub-study b) aimed to explore PCN policy implementation in areas beyond the Yorkshire and Humber and North-East regions. Opportunistic sampling and snowballing techniques, including the use of social media, were used here to recruit senior pharmacists, from across England, with responsibility for integrating PCN clinical pharmacists. Sub-study c) was primarily focused on the training and role development of clinical pharmacists who were new in post, but three of the pharmacists (in a sample of ten) were appointed or promoted to a senior pharmacy role during the study and therefore assumed some responsibility for the new clinical pharmacy workforce. In total, 17 PCNs were included across the data sets.

For peer review only

An initial topic guide was created that included questions about PCN formation and clinical pharmacy workforce integration. This was piloted with a senior pharmacist, and applied flexibly within the interviews. TM and MM carried out the interviews, all of which except one were digitally recorded and transcribed (due to a technical fault but detailed notes were taken immediately after the interview). Interview duration ranged from 42 minutes to 120 minutes. Data analysis proceeded iteratively alongside data collection to allow emerging issues of interest to be identified for further exploration. Data analysis combined the framework method(19) with complex systems modelling(20, 21). Data were organised in NVivo 12 and summarised in a Microsoft Word document. TM and MM worked together to develop and apply a coding framework to enhance the trustworthiness and credibility of the analytic process. This was applied to all data using NVIVO. A narrative summary of each interview was then created to ensure the coding process did not strip away the contextual richness of interviewee accounts(22). Summaries of key, common codes, in a form of framework analysis, allowed for cross-case comparison.

An iterative process of analysis, theme development, modelling and group discussion then ensued, involving the research team, senior academics, practitioners and PPI representatives on the project steering group. An initial set of themes, based on the data summaries, was generated by TM in a Microsoft Word document. This was refined through in-depth discussion between TM, MM and JM and further testing against the summaries and NVIVO data. The model was developed alongside this by, firstly, summarising the outcomes described by interviewees for inclusion in the model. Then, factors that appeared to contribute to the outcomes, including roles, activities and contextual moderators, were tested against the data, to develop the model. Shapes and arrows were used creatively to model contingencies and dynamic relationships, in accordance with established guidance(20, 21). The aim was not to provide a precise causal mapping of the implementation system but to provide a grounded, summary account of the roles and activities involved in local policy implementation, in order to identify candidate contributory factors for local-level outcomes across the diverse PCN settings included in the sample. An early draft of the model was presented to the project steering group and was subsequently revised, based on feedback received.

Patient and public involvement

Due to the primary focus of the study on senior staff implementation of a national policy, no patients were involved. The study sits within a research programme that features a large PPI group who were consulted throughout the research process. Patient representatives on the project steering group took part in discussions about findings.

Findings

Interview data on the early integration of the new clinical pharmacy workforce were summarised in 2 overarching themes:

1. Local organisational innovations of a national policy under conditions of uncertainty

For peer review only

2. Local multi-professional decision-making on clinical pharmacy workforce integration and initial task assignment

Theme 1. **Local organisational innovations of a national policy under conditions of uncertainty**

Although a phased implementation of the PCN package was planned (with PCNs forming first, followed by the gradual expansion of the clinical pharmacy workforce), PCNs were still forming at the time of data collection and key decisions pertaining to PCN formation extended into the integration of the clinical pharmacy workforce. Indeed, in order to qualify for funding for the new workforce, GP practices had to first form a PCN. This required the appointment of a Clinical Director and decisions about size, membership and operational model, the latter determining how the new clinical pharmacists would be employed. These decisions involved significant uncertainties, trade-offs and risks and were subject to ongoing debate.

The Clinical Director role was widely identified, by both Clinical Directors and the senior pharmacists in the sample, as being vital to building relationships across PCNs, ironing out emergent challenges (such as finding desk space for the new clinical pharmacy workforce) and making the case to primary care colleagues for participation in the PCN. The Clinical Directors within the sample were, however, new to a leadership role and reported many challenges. The publication of a significant amount of national, policy documentation to be read and implemented at speed, alongside other complex national policies, made it difficult for Clinical Directors to understand the details of what was expected locally. The process of forming a PCN was particularly challenging where GP practices were new to collaborating across organisational divides. One Clinical Director, who described their PCN's setup as being "fraught with contention", had recently resigned from post, citing workload concerns (CD-1).

Some GPs and practice staff were reported to be skeptical of the workload implications of the PCN policy because the new clinical pharmacists would have to be trained and supported in the role. Some previous, negative experiences of working with pharmacists in primary care heightened such concerns. Local skepticism could make PCN participation a hard "sell" for Clinical Directors, especially as the early impact of the PCN policy had been to increase rather than decrease workloads as had been intended:

> *I don't see any reduction in anyone's workload at the moment. If anything, we're getting busier. Its: 'We've got lots of staff but we're not sure what they're doing'. That's what they're telling me. Obviously, it's me that needs to sell that to them but it's very hard to try and convince them (CD-2)*

A further stumbling block was uncertainty about the extent that PCNs could decide on priorities for the new clinical pharmacy workforce. Some planned PCN clinical pharmacy services, notably the Structured Medication Review, were entirely new to primary care. The perception locally was that they would therefore not reduce current practice workloads, leaving little incentive to support the PCN policy:

For peer review only - http://bmjopen.bmj.com/site/about/guidelines.xhtml

> *If it's 'Would you like to use a new workforce to do additional work as well as what you're already doing?", there's no incentive there (SP-1b)*

NHS England's decision to permit a period of prolonged flexibility regarding the utilisation of the new clinical pharmacist workforce, due to the Covid-19 pandemic(23), was widely welcomed. However, there was widespread concern this would give way in favour of a performance management approach in future:

> *Unfortunately, there's a bit of uncertainty about what NHS England are going to want for their money … [attached] metrics don't necessarily align with practice priorities (SP-1)*

Some PCNs decided on operational models that implied least risk for member practices, despite the presence of trade-offs, because of the unclear benefits of the clinical pharmacy workforce. Operational models for employing this new workforce ranged from direct employment by individual GP practices or by a single, typically large general practice holding the employment contracts for all general practices within a PCN (i.e., the 'Lead Practice' model); to indirect employment via a GP Federation (i.e., a pre-existing, formal grouping of GP practices) or contracted from a private provider. While direct employment was widely seen to enhance the capacity of PCNs to manage and direct the new clinical pharmacy workforce, the risk of employment liabilities increased:

> *There's a lot of anxiety about liabilities for practices because GP partnerships are not limited liability companies … it could be a risk if you end up in an employment tribunal or something like that with risk to the partners … There was a lot of uncertainty about the structure of the networks, how legally it would work, how a contractor would work with each practice and so on, that practices felt a bit uneasy …. To be truthful, we're still negotiating how that's going to work (CD-1)*

PCNs that mapped onto pre-existing GP Federations could get established and initiate processes for recruiting the new clinical pharmacists quickly. This meant they could recruit pharmacists that they considered most suited to the role, in what was widely reported to be a tight labour market. One Clinical Director of a GP Federation-aligned PCN reported, unusually, having to "bat them away" (CD-3a). However, the GP Federation model received contrasting appraisals in the sample. Some PCN leaderships raised concerns about a lack of operational autonomy. One senior pharmacist argued that their GP Federation provided inadequate support during the integration of the clinical pharmacists while creating an "identity problem" for them once in post:

> *Unfortunately, our GP Federation isn't a strong one. They don't give any support other than payroll, and they take a management fee… It leaves people with an identity problem. Their employer is the GP Fed but it doesn't do anything other than pay their salary (SP-1b)*

The absence of an optimal operational model meant that PCN decisions about employment arrangements were subject to ongoing debate and experimentation that extended into the early utilisation of the new clinical pharmacists once recruited. While some GPs and practice managers were reported to be skeptical initially, positive

experiences of the clinical pharmacists, once in post, could increase support for the PCN policy, with subsequent implications for local choices about operational models. One Clinical Director reported that local practices were seeking to pay for additional PCN clinical pharmacists because successful, early integration had given practices more “confidence”:

> *It's given practices more confidence to almost try before they buy into it, and take on the employment risks and rights themselves (CD-3b)*

Local opinion could shift in favour of the PCN policy as a result of the new clinical pharmacists’ contributions, in some PCNs, to the Covid-19 response. One Clinical Director observed an increase in support for direct employment due to the crucial role their clinical pharmacists had served in the primary-care led vaccine programme, which was rolled out during the study period. This had, unexpectedly, presented formative opportunities for their PCN pharmacy team which contrasted with a neighbouring PCN that had decided against direct employment:

> *It became a catalyst, actually, to integrate the team into the PCN. There’s nothing else except vaccine, you eat, sleep, dream about vaccine. It would have been impossible without having a pharmacist in the PCN. It made such a difference compared to our neighbouring PCN who hasn’t had their own inhouse pharmacist (CD-2b)*

Theme 2. **Local multi-professional decision-making on clinical pharmacy workforce integration and early task assignment**

A long-term aim of the PCN policy was to implement, at scale, a patient-facing clinical pharmacy role, although there was recognition that the new clinical pharmacy workforce would take time to develop the requisite skills for this. In order to qualify for ARRS funding, newly recruited clinical pharmacists had to undertake formal training for the role, delivered by the Centre for Pharmacy Postgraduate Education (CPPE). This 18-month pathway provided them with an introduction to primary care and training in patient-facing, consultation skills. There was also an expectation that each clinical pharmacist would be assigned a GP supervisor to develop and support them in the role. However, this took place within a context of staff and service pressures and the Covid-19 pandemic. While the primary-care led vaccine programme presented formative opportunities for some PCN pharmacy teams, constraints on in-person interaction were widely held to slow the development of a patient-facing role. One senior pharmacist, who had had 18 years of experience of primary care pharmacy, warned that policymakers’ unrealistic expectations were setting up clinical pharmacists to fail:

> *From my understanding of advanced practice, I wouldn’t put myself [yet] where they’re selling the clinical pharmacists will be in 18-months’ time. You’re setting them up to fail (SPX-9)*

The senior pharmacists in the sample had roles planning PCN pharmacy services and assisting with recruitment, management and supervision. They highlighted the unique experience, knowledge and skills they brought to this role, including their understanding of primary care pharmacy and, indeed, medications, which GPs do not necessarily

For peer review only - http://bmjopen.bmj.com/site/about/guidelines.xhtml

have. The supervision they provided was considered complementary to that of GPs, focusing on medication-related questions, with GPs answering diagnostic-related questions. Having gone through a similar process to the new recruits, moreover, they considered themselves well-placed to build confidence and adapt early task assignment to each individual:

> *We decide, almost through shared decision-making with them, what level they're at, sign off their competencies, build it up slowly, give them, not boring tasks but simpler things … And then, from building your confidence, we'll develop you into the clinical pharmacist that … we want in our team (SPX-1)*

Yet, senior pharmacists' autonomy to shape the integration of the clinical pharmacists differed markedly. The extent that Clinical Directors were "pro-pharmacy" or knowledgeable of the clinical pharmacist role was reported to vary:

> *If I compare our PCN against some of the other[s] … within our locality, the Clinical Directors have been pro-pharmacy … The other Clinical Directors have struggled to grasp what a clinical pharmacist can do (SPX-8)*

Many of the senior pharmacists shared concerns about "GP led" PCNs (SPY-3b) which lacked sufficient senior pharmacy input and were purportedly employing clinical pharmacists solely as a means of reducing practice workloads rather than balancing practice priorities with strategic PCN objectives. Although many expressed sympathies with GPs about longstanding workload pressures in primary care, the senior pharmacists were concerned that, in these PCNs, the initial tasks being assigned were not aligned with the skills, experiences or aptitude of the new clinical pharmacists. Examples of early over-utilisation and under-utilisation were reported, with possible implications for staff wellbeing, retention and patient safety. For example, one senior pharmacist described how early, GP-led task assignment may have contributed to medication-related errors:

> *We've just had a massive issue switching everyone from Warfarin to NOACs [novel oral anticoagulants]. The pharmacists didn't really have any training in it, so we've now got people on NOACs that shouldn't be on NOACs. I've found two that need to back on Warfarin. So, they need to have training before they do anything and that seems to be a mismatch (SP-2)*

A more common concern was that new clinical pharmacists were being assigned basic administrative tasks, described as "all the, kind of, mop up jobs for the GP" (SPX-1). The prospect of path dependency in role development was also widely recognised, with some PCNs described as being "stuck in a rut" (SPY-2).

PCN leaderships developed contrasting approaches for resolving local tensions between PCN and GP practice priorities in task assignment. One Clinical Director had sought to integrate the clinical pharmacists closely into the general practices of their PCN. They had "over-invested" to ensure that each practice had one clinical pharmacist assigned to them such that early tasks could, to a certain extent, be adapted to each practice's requirements. Recognising the need to balance practice priorities with PCN objectives, however, they had successfully made the case for their PCN to employ a

For peer review only

senior pharmacist in an overseeing role to ensure that early tasks afforded the potential for role development in line with long-term PCN objectives. By contrast, some PCNs integrated the clinical pharmacists at PCN-level, so they were not so closely integrated into individual general practices. They could then offer input to general practices as a unified PCN pharmacy team, reducing the potential for the over- or under-utilisation of individual clinical pharmacists within practices. Both approaches were reported to have strengths and weaknesses. As with PCN choice of operational model, approaches to integrating the clinical pharmacists across PCNs and practices were subject to change as learning about the roles was acquired locally, and increasing numbers of clinical pharmacists were employed. One senior pharmacist, critical of their PCN leadership's approach, saw an opportunity to rethink this as their Clinical Director stepped down:

> *I think now is the opportunity, with this new Clinical Director coming in, to pull them out, do more training with them and change and direct the role, because they're not practice pharmacists, they're not care home pharmacists, they're not CCG pharmacists; they're PCN pharmacists (SP-2)*

The PCN policy emerging in practice

The findings encapsulate key issues relevant to the early integration of the clinical pharmacist workforce within newly forming PCNs; Figure 1 provides, in diagrammatical form, a cross-theme summary model of the implementation of this complex policy package, grounded in interviewee accounts.

Figure 1: **Early Implementation of the PCN Clinical Pharmacy Role in Newly Formed PCNs**

[Enter figure]

To the left of the model are those aspects of the national PCN policy that were observed to be stimulating local action and decision-making on the ground, notably PCN funding and the DES contract. The two overlapping circles in the middle represent the core, intertwined processes involved in local implementation: PCN formation and clinical pharmacist integration. The initial plan was for PCNs to form in the first year of the policy and for PCN pharmacy teams to expand thereafter: the overlap, along with the double-ended, curved arrows linking the circles, indicate that PCNs were not developing in such a linear process. Rather, PCNs were still being formed more than 2 years into policy implementation and crucial decisions about operating models extended long into the process of integrating the clinical pharmacists into PCNs. Furthermore, local learning about the possible contribution of clinical pharmacists could lead to decisions pertaining to PCN operational models being revisited, with a view to more optimal integration in future.

The core outcomes, at this stage of PCN policy implementation, are summarised to the right of model: outcomes were categorised as optimal or suboptimal, based on interviewees' evaluations of clinical pharmacist integration. The double-ended arrows between the outcomes and the circle representing clinical pharmacist integration raise the possibility of feedback loops where an initial success paves the way for further

For peer review only - http://bmjopen.bmj.com/site/about/guidelines.xhtml

successes as PCN members see the potential of the new workforce. The converse is also illustrated, as early failures are constraining and invite path dependency in role development. Below, the ‘moderating factors’ are candidate explanatory factors, based on this analysis, for variations in the outcomes across the PCNs in the sample. The external threats identified at the top of the model comprise factors reported to impede progress across PCNs generally, although more advanced PCN pharmacy teams could mitigate their effects or, as in the case of the primary-care led vaccine programme, create an opportunity out of them.

Discussion

This paper contributes to scholarship on NHS primary care restructuring and the “mid-level” professional roles that are emerging globally(8,9). In particular, it highlights the challenges involved in scaling up new roles across a health system, here a primary-care, clinical pharmacy role, being implemented via PCNs, as part of a major policy effort to embed elements of international best practice in the English NHS. The contribution includes examples provided, by senior pharmacists, of clinical pharmacists being over- and under-utilised in their early task assignment, with the allocation of excessive basic administrative tasks being a widespread concern. The potential for path dependency in role development, also referenced in the analysis, implies a significant challenge to long-term PCN objectives. This is consistent with a recent report from The King’s Fund, which highlights considerable variation in emerging PCN practice on the ground, amidst a lack of shared, local understanding about the purpose of the new PCN roles(24). This emerging picture of undesirable variation in PCN policy implementation suggests the cogency of concerns voiced earlier on in the policy cycle that policymakers were being overly optimistic about what could be achieved in a challenging environment(16,25).

Indeed, Checkland et al’s interview study of national policymakers and stakeholders in the PCN policy in its formative stage revealed contrasting policy objectives that, the authors warned, were likely to complicate implementation(16). Notable among these was a tension between the objective of supporting primary care via investment and the objective of facilitating the design and delivery of new services across organisational boundaries. Checkland et al(16) concluded that stronger temporal sequencing of implementation, focusing on the development of primary care first before the delivery of new services, would maximise the likelihood of success. Our findings attest to the clear sightedness of Checkland et al’s analysis(16). Although a phased implementation of the PCN package was planned (with PCNs to form first, before integrating the clinical pharmacy workforce), we found that processes underpinning PCN formation and clinical pharmacist workforce integration were closely intertwined and taking place under considerable workforce pressures. Somewhat ironically, Covid-19 enforced a stronger version of temporal sequencing, similar to that recommended by Checkland et al(16), as the delivery of PCN services was paused such that a new workforce was available to assist in the primary care led vaccine programme(9). Our findings suggest that PCNs have had varying degrees of success in integrating the clinical pharmacist workforce under these conditions however. Immediate research and policy implications of the analysis are discussed next, followed by implications for wider debates about NHS governance:

Research implications – The unfolding course of events in restructuring primary care and optimising the contribution of the expanded clinical pharmacist workforce deserve to be studied closely, as do other national policy innovations largely or entirely contingent upon local priorities for delivery. The key research implications here concern the workforce development needs of clinical pharmacists, how these are met in practice, and with what impacts on the roles that emerge.

Policy implications – A comprehensive package of support, as outlined in The King's Fund report(24), is required for PCNs. This will require the new partnership structures known as Integrated Care Systems, also still forming, to provide organisational and leadership skills development and service redesign support. This study has also highlighted a particular need for a clearer policy and professional framework for senior PCN pharmacists. In addition, an extension of the ARRS funding, beyond the five years planned, would provide struggling PCNs with more time, and may alleviate some of the concern about employment liabilities. A nuanced and differentiated approach to DES service implementation is urgently required going forward, to take into account the very local context of the capacity for PCN innovation.

Governance implications – The decision to deploy a contract as the primary mechanism to develop primary care requires some reflection. Advanced understanding of the limitations of contracts in healthcare delivery is long established(26). Contracts operate via centralised agreements and lack corrective mechanisms, making them poorly suited to the supportive and nuanced approach to DES service implementation that is necessary for the PCN policy to progress in the direction of fulfilling its aims. Issues pertaining to PCN formation and employment contracts also invite consideration of transaction cost literatures which highlight potential savings from the direct employment of health professionals via statutory bodies(27). An alternative, public sector administration approach to the development of primary care would be to integrate GP practices within the NHS and to employ primary care professionals directly(28). A piloting approach(29) could also be adopted in future to test and evaluate new primary care roles and services, with policy frameworks developed which are informed by well understood best practice.

For peer review only

Footnotes

Ethics approval

The study received research ethics approval (REC reference 20/HRA/1482).

Funding

This research was funded by the National Institute for Health Research [NIHR] PGfAR [RP-PG-0216-20002]. The views expressed are those of the authors and not necessarily those of the NIHR or the Department of Health and Social Care.

Patient consent for publication

None required

Author contributions

JM and MM designed the study. TM and MM conducted the interviews and analysed the data. TM conceptualised and led the write up of the paper, to which all authors made substantial contributions.

Competing interests

The Authors declare that there is no conflict of interest

Data availability statement

No data are available. This study has not received ethical approval to share confidential data with any third party other than the study research team.

For peer review only

Bibliography

1. WHO UN. A vision for primary health care in the 21st century [Internet]. World Health Organisation and United Nations Children's Fund. 2018 [cited 2021 Nov 30]. Available from: https://www.who.int/westernpacific/health-topics/primary-health-care

2. Roland M, Guthrie B, Thomé DC. Primary medical care in the United Kingdom. J Am Board Fam Med JABFM. 2012;25 Suppl 1:S6-11.

3. O'Cathain A, Musson G, Munro J. Shifting Services from Secondary to Primary Care: Stakeholders' Views of the Barriers. J Health Serv Res Policy. 1999 Jul 1;4(3):154–60.

4. Doran T, Roland M. Lessons from major initiatives to improve primary care in the United Kingdom. Health Aff Proj Hope. 2010 May;29(5):1023–9.

5. Laurant M, Reeves D, Hermens R, Braspenning J, Grol R, Sibbald B. Substitution of doctors by nurses in primary care. Cochrane Database Syst Rev. 2005 Apr 18;(2):CD001271.

6. Bradley F, Seston E, Mannall C, Cutts C. Evolution of the general practice pharmacist's role in England: a longitudinal study. Br J Gen Pract J R Coll Gen Pract. 2018 Oct;68(675):e727–34.

7. Drennan VM, Collins L, Allan H, Brimblecombe N, Halter M, Taylor F. Are advanced clinical practice roles in England's National Health Service a remedy for workforce problems? A qualitative study of senior staff perspectives. J Health Serv Res Policy. 2021 Aug 10;13558196211036728.

8. WHO. Global strategy on human resources for health: workforce 2030 [Internet]. World Health Organisation. 2016 [cited 2021 Nov 30]. Available from: https://apps.who.int/iris/bitstream/handle/10665/250368/9789241511131-eng.pdf?sequence=1&isAllowed=y

9. Stewart D, Madden M, Davies P, Whittlesea C, McCambridge J. Structured medication reviews: origins, implementation, evidence, and prospects. Br J Gen Pract. 2021;71(709):340–1.

10. Baird B, Charles A, Honeyman M, Maguire D, Das P. Understanding pressures in general practice. Kings Fund. 2016;100.

11. NHS. NHS long term plan [Internet]. NHS England. 2019 [cited 2021 Nov 30]. Available from: https://www.longtermplan.nhs.uk

12. BMA. The primary care network handbook. British Medical Association; 2020.

For peer review only

13. Leslie M, Khayatzadeh-Mahani A, Birdsell J, Forest PG, Henderson R, Gray RP, et al. An implementation history of primary health care transformation: Alberta's primary care networks and the people, time and culture of change. BMC Fam Pract. 2020 Dec 5;21(1):258.

14. Nuffield Trust. Primary care for the 21st century: learning from New Zealand's independent practitioner associations. Nuffield Trust; 2012.

15. Silcock J, Raynor DKT, Petty D. The organisation and development of primary care pharmacy in the United Kingdom. Health Policy Amst Neth. 2004 Feb;67(2):207–14.

16. Checkland K, Hammond J, Warwick-Giles L, Bailey S. Exploring the multiple policy objectives for primary care networks: a qualitative interview study with national policy stakeholders. BMJ Open. 2020 Jul 1;10(7):e038398.

17. Morris S, Madden M, Gough B, Atkin K, Mccambridge J. Missing in action: Insights from an exploratory ethnographic observation study of alcohol in everyday UK community pharmacy practice. Drug Alcohol Rev. 2019 Jul;38(5):561–8.

18. Stewart D, Madden M, Van Dongen A, Watson M, Morris S, Whittlesea C, et al. Process study within a pilot cluster randomised trial in community pharmacy: An exploration of pharmacist readiness for research. Res Soc Adm Pharm RSAP. 2021 Oct;17(10):1750–7.

19. Gale NK, Heath G, Cameron E, Rashid S, Redwood S. Using the framework method for the analysis of qualitative data in multi-disciplinary health research. BMC Med Res Methodol. 2013 Sep 18;13(1):117.

20. Mills T, Lawton R, Sheard L. Advancing complexity science in healthcare research: the logic of logic models. BMC Med Res Methodol. 2019 Mar 12;19(1):55.

21. Mills T, Shannon R, O'Hara J, Lawton R, Sheard L. Development of a 'real-world' logic model through testing the feasibility of a complex healthcare intervention: the challenge of reconciling scalability and context-sensitivity. Evaluation. 2022 Jan 22;13563890211068868.

22. Ayres L, Kavanaugh K, Knafl KA. Within-Case and Across-Case Approaches to Qualitative Data Analysis. Qual Health Res. 2003 Jul 1;13(6):871–83.

23. NHS. Structured medication reviews and medicines optimisation: guidance. NHS Engl [Internet]. 2020; Available from: https://www.england.nhs.uk/wp-content/uploads/2020/09/SMR-Spec-Guidance-2020-21-FINAL-.pdf

24. Baird B, Lamming L, Bhatt R, Beech J, Dale V. Integrating additional roles into primary care networks. Kings Fund. 2022;

For peer review only

25. Pettigrew L, Mays N. Primary care networks: the risk of “mission creep” calls for focused ambition. BMJ. 2019;367:l5978.

26. Allen P. A socio-legal and economic analysis of contracting in the NHS internal market using a case study of contracting for district nursing. Soc Sci Med. 2002;54(2):255–66.

27. Hodgson GM. Towards an alternative economics of health care. Health Econ Policy Law. 2009 Jan;4(Pt 1):99–114.

28. Leys C. The English NHS: from market failure to trust, professionalism and democracy. Soundings. 2017;64:11–40.

29. Checkland K, Hammond J, Coleman A, Macinnes J, Mikelyte R, Croke S, et al. “Success” in policy piloting: Process, programs, and politics. Public Adm [Internet]. [cited 2021 Dec 1];n/a(n/a). Available from: https://onlinelibrary.wiley.com/doi/abs/10.1111/padm.12790

For peer review only

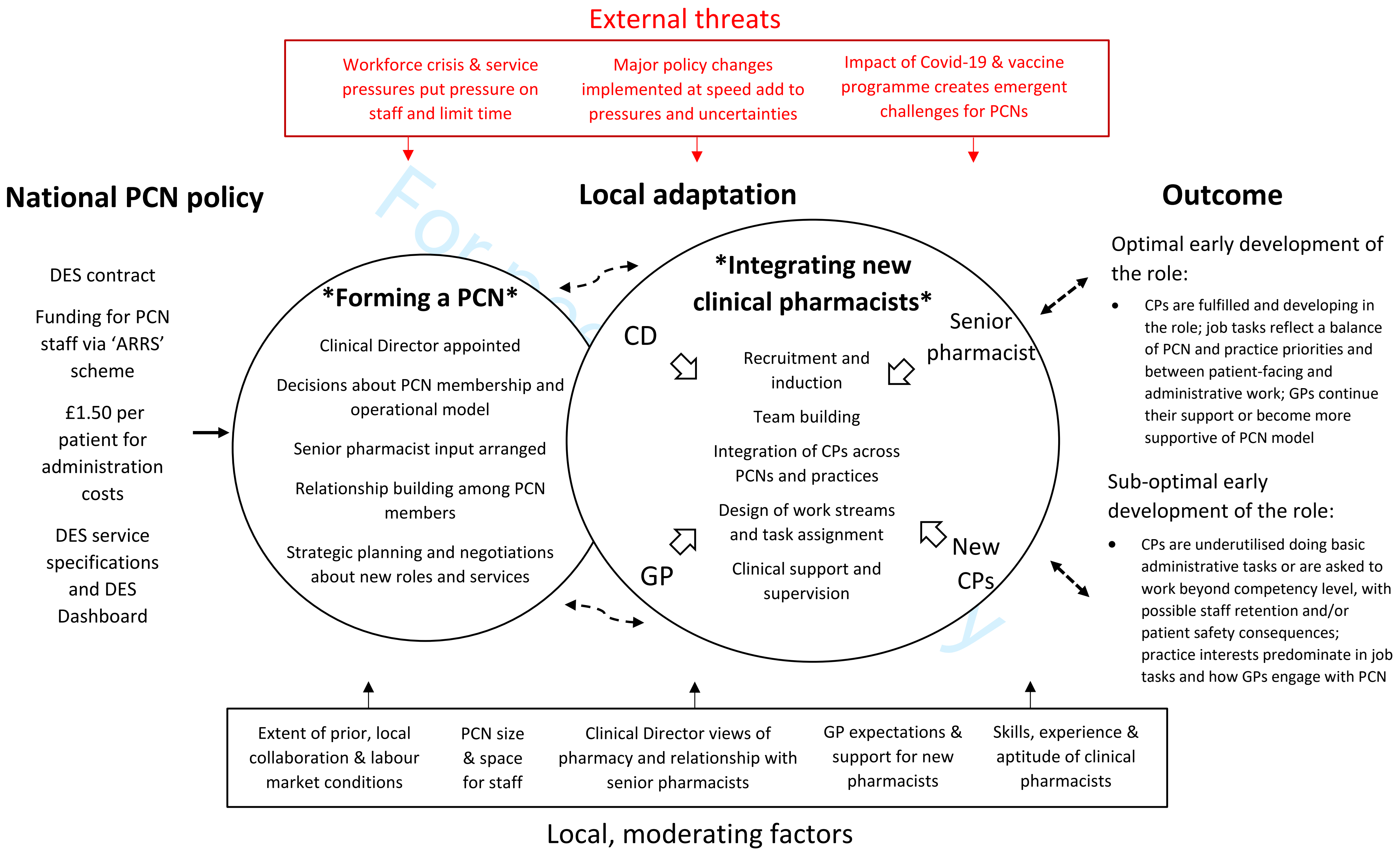
External threats
Workforce crisis & service pressures put pressure on staff and limit time
Major policy changes implemented at speed add to pressures and uncertainties
Impact of Covid-19 & vaccine programme creates emergent challenges for PCNs
National PCN policy
DES contract
Funding for PCN staff via 'ARRS' scheme
£1.50 per patient for administration costs
DES service specifications and DES Dashboard
Local adaptation
Forming a PCN
Clinical Director appointed
Decisions about PCN membership and operational model
Senior pharmacist input arranged
Relationship building among PCN members
Strategic planning and negotiations about new roles and services
Integrating new clinical pharmacists
CD
Senior pharmacist
GP
New CPs
Recruitment and induction
Team building
Integration of CPs across PCNs and practices
Design of work streams and task assignment
Clinical support and supervision
Outcome
Optimal early development of the role:
CPs are fulfilled and developing in the role; job tasks reflect a balance of PCN and practice priorities and between patient-facing and administrative work; GPs continue their support or become more supportive of PCN model
Sub-optimal early development of the role:
CPs are underutilised doing basic administrative tasks or are asked to work beyond competency level, with possible staff retention and/or patient safety consequences; practice interests predominate in job tasks and how GPs engage with PCN
Extent of prior, local collaboration & labour market conditions
PCN size & space for staff
Clinical Director views of pharmacy and relationship with senior pharmacists
GP expectations & support for new pharmacists
Skills, experience & aptitude of clinical pharmacists
Local, moderating factors

Standards for Reporting Qualitative Research (SRQR)*

http://www.equator-network.org/reporting-guidelines/srqr/

Title and abstract

	Page/line no(s).
**Title** - Concise description of the nature and topic of the study Identifying the study as qualitative or indicating the approach (e.g., ethnography, grounded theory) or data collection methods (e.g., interview, focus group) is recommended	See page 1
**Abstract** - Summary of key elements of the study using the abstract format of the intended publication; typically includes background, purpose, methods, results, and conclusions	See page 2 – we have followed BMJ Open requirements in writing the abstract

Introduction

**Problem formulation** - Description and significance of the problem/phenomenon studied; review of relevant theory and empirical work; problem statement	We introduce the problem and review relevant research in the introduction. This includes reference to Checkland et al (2020)'s qualitative work, in BMJ Open – see page 4
**Purpose or research question** - Purpose of the study and specific objectives or questions	We summarise the research purpose in the final sentences of the introduction – see page 5

Methods

**Qualitative approach and research paradigm** - Qualitative approach (e.g., ethnography, grounded theory, case study, phenomenology, narrative research) and guiding theory if appropriate; identifying the research paradigm (e.g., postpositivist, constructivist/ interpretivist) is also recommended; rationale**	We discuss our use of the framework method and complex systems modelling – see page 6

For peer review only

For peer review only - http://bmjopen.bmj.com/site/about/guidelines.xhtml

**Researcher characteristics and reflexivity** - Researchers' characteristics that may influence the research, including personal attributes, qualifications/experience, relationship with participants, assumptions, and/or presuppositions; potential or actual interaction between researchers' characteristics and the research questions, approach, methods, results, and/or transferability	The start of the methods section makes clear that the research was undertaken as part of a broader research programme that is assessing the potential for pharmacists to discuss alcohol within medicine reviews. Links to the research team's publications are provided – see page 5
**Context** - Setting/site and salient contextual factors; rationale**	We discuss salient contextual factors, notably the challenges of sampling diverse PCNs when they were still forming and changing in composition – see page 5
**Sampling strategy** - How and why research participants, documents, or events were selected; criteria for deciding when no further sampling was necessary (e.g., sampling saturation); rationale**	We describe the sampling strategy through pages 5 and 6
**Ethical issues pertaining to human subjects** - Documentation of approval by an appropriate ethics review board and participant consent, or explanation for lack thereof; other confidentiality and data security issues	We address issues about informed consent and include the REC reference in the footnotes – see page 14
**Data collection methods** - Types of data collected; details of data collection procedures including (as appropriate) start and stop dates of data collection and analysis, iterative process, triangulation of sources/methods, and modification of procedures in response to evolving study findings; rationale**	We describe the data collection methods in detail through the methods section – see page 6

For peer review only - http://bmjopen.bmj.com/site/about/guidelines.xhtml

**Data collection instruments and technologies** - Description of instruments (e.g., interview guides, questionnaires) and devices (e.g., audio recorders) used for data collection; if/how the instrument(s) changed over the course of the study	We describe the data collection instruments and technologies in detail through the methods section – see page 6
**Units of study** - Number and relevant characteristics of participants, documents, or events included in the study; level of participation (could be reported in results)	We describe the sample and the level of participation of the participants – see page 5
**Data processing** - Methods for processing data prior to and during analysis, including transcription, data entry, data management and security, verification of data integrity, data coding, and anonymization/de-identification of excerpts	We describe data processes, including an outline of the analytic process from data collection to the analysis stage, with reference to transcription and data coding – see page 6
**Data analysis** - Process by which inferences, themes, etc., were identified and developed, including the researchers involved in data analysis; usually references a specific paradigm or approach; rationale**	The analytic process through which the thematic findings and model were derived is described – see page 6
**Techniques to enhance trustworthiness** - Techniques to enhance trustworthiness and credibility of data analysis (e.g., member checking, audit trail, triangulation); rationale**	We outline our approach to ensuring trustworthiness on page 6

Results/findings

**Synthesis and interpretation** - Main findings (e.g., interpretations, inferences, and themes); might include development of a theory or model, or integration with prior research or theory	The main thematic findings are presented first. This is followed by the presentation of the complex systems model

For peer review only - http://bmjopen.bmj.com/site/about/guidelines.xhtml

	which brings together issues and factors described in the themes – see pages 6 to 12
**Links to empirical data** - Evidence (e.g., quotes, field notes, text excerpts, photographs) to substantiate analytic findings	Quotes from the empirical data are threaded through the findings section – see pages 6 to 11

Discussion

**Integration with prior work, implications, transferability, and contribution(s) to the field -** Short summary of main findings; explanation of how findings and conclusions connect to, support, elaborate on, or challenge conclusions of earlier scholarship; discussion of scope of application/generalizability; identification of unique contribution(s) to scholarship in a discipline or field	We draw out the implications of the research in the discussion section and discuss prior research on the topic. We include research and policy implications – see pages 12 to 13
**Limitations** - Trustworthiness and limitations of findings	We discuss the study's strengths and limitations in line with BMJ Open requirements – see page 3

Other

**Conflicts of interest** - Potential sources of influence or perceived influence on study conduct and conclusions; how these were managed	A statement on conflicts of interest is included – see page 14
**Funding** - Sources of funding and other support; role of funders in data collection, interpretation, and reporting	A statement of funding is also included – see page 14

For peer review only

*The authors created the SRQR by searching the literature to identify guidelines, reporting standards, and critical appraisal criteria for qualitative research; reviewing the reference lists of retrieved sources; and contacting experts to gain feedback. The SRQR aims to improve the transparency of all aspects of qualitative research by providing clear standards for reporting qualitative research.

**The rationale should briefly discuss the justification for choosing that theory, approach, method, or technique rather than other options available, the assumptions and limitations implicit in those choices, and how those choices influence study conclusions and transferability. As appropriate, the rationale for several items might be discussed together.

Reference:

O'Brien BC, Harris IB, Beckman TJ, Reed DA, Cook DA. **Standards for reporting qualitative research: a synthesis of recommendations.** *Academic Medicine*, Vol. 89, No. 9 / Sept 2014 DOI: 10.1097/ACM.0000000000000388

For peer review only

For peer review only - http://bmjopen.bmj.com/site/about/guidelines.xhtml

BMJ Open

The integration of a clinical pharmacist workforce into newly forming Primary Care Networks: a qualitatively-driven, complex systems analysis

Journal:	*BMJ Open*
Manuscript ID	bmjopen-2022-066025.R1
Article Type:	Original research
Date Submitted by the Author:	12-Sep-2022
Complete List of Authors:	Mills, Thomas; University of York; London South Bank University<br>Madden, Mary; University of York,<br>Stewart, Duncan; London Metropolitan University,<br>Gough, Brendan; Leeds Beckett University<br>McCambridge, Jim; University of York
**Primary Subject Heading**:	Health policy
Secondary Subject Heading:	Health services research
Keywords:	Health policy < HEALTH SERVICES ADMINISTRATION & MANAGEMENT, PRIMARY CARE, QUALITATIVE RESEARCH, Organisation of health services < HEALTH SERVICES ADMINISTRATION & MANAGEMENT, Change management < HEALTH SERVICES ADMINISTRATION & MANAGEMENT, International health services < HEALTH SERVICES ADMINISTRATION & MANAGEMENT

SCHOLARONE™
Manuscripts

I, the Submitting Author has the right to grant and does grant on behalf of all authors of the Work (as defined in the below author licence), an exclusive licence and/or a non-exclusive licence for contributions from authors who are: i) UK Crown employees; ii) where BMJ has agreed a CC-BY licence shall apply, and/or iii) in accordance with the terms applicable for US Federal Government officers or employees acting as part of their official duties; on a worldwide, perpetual, irrevocable, royalty-free basis to BMJ Publishing Group Ltd ("BMJ") its licensees and where the relevant Journal is co-owned by BMJ to the co-owners of the Journal, to publish the Work in this journal and any other BMJ products and to exploit all rights, as set out in our licence.

The Submitting Author accepts and understands that any supply made under these terms is made by BMJ to the Submitting Author unless you are acting as an employee on behalf of your employer or a postgraduate student of an affiliated institution which is paying any applicable article publishing charge ("APC") for Open Access articles. Where the Submitting Author wishes to make the Work available on an Open Access basis (and intends to pay the relevant APC), the terms of reuse of such Open Access shall be governed by a Creative Commons licence – details of these licences and which Creative Commons licence will apply to this Work are set out in our licence referred to above.

Other than as permitted in any relevant BMJ Author's Self Archiving Policies, I confirm this Work has not been accepted for publication elsewhere, is not being considered for publication elsewhere and does not duplicate material already published. I confirm all authors consent to publication of this Work and authorise the granting of this licence.

For peer review only

The integration of a clinical pharmacist workforce into newly forming Primary Care Networks: a qualitatively-driven, complex systems analysis

Title page

Author list:

Dr Thomas Mills, University of York, York, YO10 5DD

Dr Mary Madden (Associate Professor), University of York, York, YO10 5DD

Professor Duncan Stewart, London Metropolitan University, 166-220 Holloway Rd, London N7 8DB

Professor Brendan Gough, Leeds Beckett University, Leeds LS1 3HE

Professor Jim McCambridge, University of York, York, YO10 5DD

Corresponding author details:

Dr Thomas Mills

University of York, York, YO10 5DD

Email: millst3@lsbu.ac.uk

TEL: 07806497970

Fax: +44 (0)1904 32 3433

Key terms: Primary Health Care, General Practice, Pharmacy, Qualitative Research, Complex Systems Modelling

Word count: 5157

For peer review only

Abstract

Objective

The introduction of a new clinical pharmacist workforce via Primary Care Networks (PCNs) is a recent national policy development in the National Health Service (NHS) in England. This study elicits the perspectives of people with responsibility for local implementation of this national policy package. Attention to local delivery is necessary to understand the contextual factors shaping the integration of the new clinical pharmacy workforce, and thus can be expected to influence future role development.

Design

A qualitative, interview study

Setting and participants

PCN Clinical Directors and senior pharmacists across 17 PCNs in England (n=28)

Analysis

Interviews were transcribed, coded and organised using the framework method. Thematic analysis and complex systems modelling were then undertaken iteratively to develop the themes.

Results

Findings were organised into two overarching themes: 1) Local organisational innovations of a national policy under conditions of uncertainty; 2) Local multi-professional decision-making on clinical pharmacy workforce integration and initial task assignment. Although a phased implementation of the PCN package was planned, the findings suggest that processes of PCN formation and clinical pharmacist workforce integration were closely intertwined, with underpinning decisions taking place under conditions of considerable uncertainty and workforce pressures.

Conclusions

National policy decisions that required GPs to form PCNs at the same time as they integrated a new workforce risked undermining the potential of both PCNs and the new workforce. PCNs require time and support to fully form and integrate clinical pharmacists if successful role development is to occur. Efforts to incentivise delivery of PCN pharmacy services in future must be responsive to local capacity.

For peer review only

Strengths and Limitations

- The present study represents a rigorous and timely qualitative investigation of the early integration of a new clinical pharmacist workforce into primary care that is anticipated to take on a significant, patient-facing role in future.
- The sampling approach allowed the research to capture insights on the new clinical pharmacy workforce across 17 diverse PCNs in England.
- While the diversity of PCNs provided broad brush insight, a case study approach that focused on a smaller number of PCNs more in depth could provide deeper lessons.
- The principal study focus on the clinical pharmacist workforce means that the study does not explore other developments that are being implemented as part of the PCN policy package.
- The study focus on senior staff (PCN Clinical Directors and senior pharmacists) could also be complemented, in future, by the qualitative study of the perspectives of the newly integrated workforce itself. Broadening the sample to include other general practice staff, e.g., practice managers and nursing staff, would also allow exploration of diverse perspectives and multidisciplinary working.

For peer review only

Introduction

Reform efforts globally have sought to develop the primary care sector with the underpinning idea that the sector is vital to contemporary healthcare challenges(1). In the UK, a range of institutional reforms have, over the past four decades, been enacted to transform the traditional set-up of small private business partnerships run by General Practitioners (GPs). GPs have traditionally been contracted by the NHS to provide a range of services to a local population and coordinate patient care, including referrals to hospital-based specialist care. Reforms to this set-up have included attempts to enhance the role of GPs in healthcare commissioning and new models of service delivery that have transferred services out of hospitals into primary care(2, 3, 4). There have also been efforts to address shortfalls in the numbers of GPs and meet new healthcare challenges, including facing the increasing demand and complexity of patient needs, through the integration and development of new primary care roles(5, 6, 7). This reflects a broader task shifting to "mid-level" professionals who are educated and trained to a level where they can undertake some of the activities of GPs and specialists within a prescribed scope of practice(7, 8). In primary care, some GP work tasks and responsibilities have shifted to nursing staff(5) and, more recently, clinical pharmacists(6, 9). Such developments occur within ongoing contexts of reorganisations, funding constraints and other pressures(10).

The NHS Long Term Plan, published in January 2019, featured a new funding settlement for primary care (with projected funding to be £4.5 billion higher in 2023/24 than in 2019/20) and plans for new staff roles and services(11, 12). In order to qualify for new funding, GPs were to collaborate with neighbouring practices to form Primary Care Networks (PCNs)(11, 12). The aim was to expand the primary care workforce and enhance patient care through new and improved services, based on aspects of Canadian(13) and New Zealand(14) primary care systems. A new national contract, the Network Contract Directed Enhanced Services (known colloquially as 'the DES'), was introduced on the 1st of July 2019. This facilitated access to funds for new healthcare roles via the Additional Roles Reimbursement Scheme (ARRS), including social prescribing link workers, physiotherapists, paramedics and pharmacists. 1,250 PCNs were formed across England in the first year of the policy, each serving around 30-50,000 patients(12).

Existing research in PCN policy implementation reveals a contrasting picture of early progress, with governance arrangements established and new staff quickly recruited, amidst significant "organisational fragility"(15). A National Institute for Health Research funded study of PCN formation found considerable challenges on set-up due to a short policy time frame and the complexities involved in collaborating across practice boundaries(16). Qualitative research in this emerging field has explored the consistency of national PCN policy objectives(17), GP views and early experiences of the PCN policy(18), local commissioner support(19) and, most recently, the integration of new healthcare roles into PCNs(20). Given that a central PCN objective is to expand the primary care workforce, there is a clear research need for further research into the integration of PCN healthcare staff and early role development.

For peer review only

This study presents a qualitative investigation of the early integration of a clinical pharmacy workforce into newly forming PCNs. Clinical pharmacists are a key example of a “mid-level” professional group(7, 8). Historically, pharmacists in the UK have mainly operated in secondary care hospitals or in community pharmacy, providing prescription dispensing services and, more recently, over-the-counter advice(21). Numbers of pharmacists in GP practices increased during the 1990s via the GP Fundholding scheme and through the 2000s, but the absence of a national workforce strategy resulted in significant diversity, in terms of numbers and roles, across GP practices(21, 6). A key long-term aim of PCN policy was to implement nationally a patient-facing clinical pharmacy role, following an initial pilot in 2016(6): PCNs were permitted to employ a single clinical pharmacist in their first year of formation (2019), with this number expected to rise to five clinical pharmacists per PCN by 2024(12). The research aim was to investigate how the PCN policy package was being implemented locally and to develop understanding of the contextual factors that were enabling or constraining the integration of the new clinical pharmacy workforce. A complex systems perspective was adopted that conceives of public policies as multi-layered systems characterised by non-linearity, emergence, feedback and adaptations across national, regional and local levels(22, 23). We sought to explore policy emergence and adaptation in diverse PCNs across England using complex systems modelling techniques to model emergent pathways and local-level outcomes(24, 25).

Methods

A range of exploratory studies were previously undertaken to assess the potential of the pharmacist role with a view to incorporating attention to alcohol within medicine reviews(9, 26, 27). These studies featured within a research programme that started in community pharmacy and then moved into general practice as the new clinical pharmacist workforce was recruited there, via PCNs. This guided the development of this study, including research questions and approach, as we sought to understand and assess the new clinical pharmacy workforce for its suitability to take part in a research trial. The study included interviews with senior staff with responsibility for integrating the clinical pharmacy workforce into newly forming PCNs. The interviews took place between March 2020, a year after PCNs were established, through to September 2021, providing a snapshot of this aspect of the national policy package. Three sets of interviews were undertaken, creating three sub-study datasets:

a) Twelve semi-structured interviews with seven senior PCN staff (three GP Clinical Directors and four senior PCN pharmacists), between March 2020 and September 2021, in six PCNs based in the Yorkshire and Humber and North-East regions of England (i.e., two interviews one year apart to explore developments over time, although one participant changed role and declined the second interview). Quotation codes: CD (Clinical Director) and SP (senior pharmacist); the addition of b indicates second interview.

b) 10 one-off semi-structured interviews between March 2021 and August 2021 with 10 pharmacists in 10 PCNs who were already working in primary care prior to the formation of PCNs and were established in a senior role (either in a GP practice or a PCN) during the transition. Quotation codes: SPX.

For peer review only

c) Six semi-structured interviews conducted between September 2020 and June 2021 with three newly appointed senior pharmacists across 4 PCNs (i.e., two interviews one year apart to explore developments over time; one interviewee switched to a different PCN during the study). Quotation codes: SPY; the addition of b indicates second interview.

The sampling strategy for sub-study a) was pragmatic and opportunistic: we initially sought diversity of PCNs, in terms of operational models and patient populations, but this was not possible to ensure because PCNs were still forming. Changing PCN characteristics were therefore explored through the interviews. The research team utilised a combination of existing PCN contacts and new contacts, established via telephone calls to GP practices, to recruit Clinical Directors and senior pharmacists into the sub-study. Sub-study b participants were recruited from PCNs across England to broaden the focus beyond the Yorkshire and Humber and North-East regions. Opportunistic sampling and snowballing techniques were used here. A leaflet describing the study was distributed via national pharmacy organisations and on social media, which included an invite to contact the research team. Sub-study c) included new clinical pharmacists who were recruited via the contacts developed through sub-studies a and b. Sub-study c primarily focused on new clinical pharmacists' early training and role but three of the pharmacists (in a sample of ten) were appointed or promoted to a senior pharmacy position during the study and therefore assumed some responsibility for the new clinical pharmacy workforce: data pertaining to PCN formation and clinical pharmacist integration were therefore incorporated into the analysis. All interviewees provided written consent, following an initial contact with the research team, during which the study was discussed and participants were screened for eligibility. In total, 28 senior PCN staff were interviewed across 17 PCNs.

An initial topic guide was created that included questions about PCN formation, clinical pharmacy workforce integration and new, PCN clinical pharmacy services (see supplementary file). This was piloted with a senior pharmacist, and applied flexibly within the interviews. TM and MM undertook all interviews separately, all except one of which were digitally recorded and transcribed (due to a technical fault but detailed notes were taken immediately after the interview). Interview duration ranged from 42 minutes to 120 minutes. Data analysis proceeded iteratively alongside data collection to allow emerging issues of interest to be identified for further exploration. Data analysis combined the framework method(28) with complex systems modelling(24, 25). Data were organised in NVivo 12 and summarised in a Microsoft Word document. TM and MM worked together to develop and apply a coding framework, cross checking and engaging in consensus discussion throughout, to ensure the trustworthiness and credibility of the analytic process. The coding framework was applied to all data using NVivo. A narrative summary of each interview was created to ensure the coding process did not strip away the contextual richness of interviewee accounts(29). Codes that were identified as significant to PCN formation and clinical pharmacist role development were summarised across the dataset, in a form of framework analysis.

An iterative process of analysis, theme development, modelling and group discussion then ensued, involving the research team, senior academics, practitioners and patient representatives on the project steering group. An initial set of themes, based on the

For peer review only - http://bmjopen.bmj.com/site/about/guidelines.xhtml

data summaries, was generated by TM in a Microsoft Word document. This was refined through cross-case comparison, in-depth discussion between TM, MM and JM and further testing against the summaries and NVivo data. The model was developed alongside this by, firstly, summarising the outcomes described by interviewees for inclusion in the model. Then, factors that appeared to contribute to the outcomes were identified and tested against the data, to develop the model. Shapes and arrows were used creatively to model contingencies and dynamic relationships, in accordance with established guidance(24, 25). The aim was not to provide a precise causal mapping of the implementation system but to provide a grounded, summary account of the roles, activities and contributory factors for local-level outcomes across the diverse PCN settings included in the sample. An early draft of the model was presented to the project steering group and was subsequently revised, based on feedback received.

Patient and public involvement

Due to the primary focus of the study on senior staff implementation of a national policy, no patients were recruited. The study sits within a research programme that features an experienced Patient and Public Involvement (PPI) group who were consulted throughout the research process(30). Patient representatives on the project steering group took part in discussions about findings.

Findings

Interview data on the early integration of the new clinical pharmacy workforce were summarised in 2 overarching themes:

1. Local organisational innovations of a national policy under conditions of uncertainty
2. Local multi-professional decision-making on clinical pharmacy workforce integration and initial task assignment

Theme 1. **Local organisational innovations of a national policy under conditions of uncertainty**

Although a phased implementation of the PCN package was planned (with PCNs forming first, followed by the gradual expansion of the clinical pharmacy workforce), PCNs were still forming at the time of data collection and key decisions pertaining to PCN formation extended into the integration of the clinical pharmacy workforce. Indeed, in order to qualify for funding for the new workforce, GP practices had to first form a PCN. This required the appointment of a Clinical Director and decisions about size, membership and operational model, the latter determining how the new clinical pharmacists would be employed. These decisions involved significant uncertainties, trade-offs and risks and were subject to ongoing debate.

The Clinical Director role was widely identified, by both Clinical Directors and the senior pharmacists in the sample, as being vital to building relationships across PCNs, ironing out emergent challenges (such as finding desk space for the new clinical pharmacy

For peer review only - http://bmjopen.bmj.com/site/about/guidelines.xhtml

workforce) and making the case to primary care colleagues for participation in the PCN. The Clinical Directors within the sample were, however, new to a leadership role and reported many challenges. The publication of a significant amount of national, policy documentation to be read and implemented at speed, alongside other complex national policies, made it difficult for Clinical Directors to understand the details of what was expected locally. The process of forming a PCN was particularly challenging where GP practices were new to collaborating across organisational divides. One Clinical Director, who described their PCN's setup as being "fraught with contention", had recently resigned from post, citing workload concerns (CD-1).

Some GPs and practice staff were reported to be skeptical of the workload implications of the PCN policy because the new clinical pharmacists would have to be trained and supported in the role. Some previous, negative experiences of working with pharmacists in primary care heightened such concerns. Local skepticism could make PCN participation a hard "sell" for Clinical Directors, especially as the early impact of the PCN policy had been to increase rather than decrease workloads as had been intended:

> *I don't see any reduction in anyone's workload at the moment. If anything, we're getting busier. Its: 'We've got lots of staff but we're not sure what they're doing'. That's what they're telling me. Obviously, it's me that needs to sell that to them but it's very hard to try and convince them (CD-2)*

A further stumbling block was uncertainty about the extent that PCNs could decide on priorities for the new clinical pharmacy workforce. Some planned PCN clinical pharmacy services, notably the Structured Medication Review, were entirely new to primary care. The perception locally was that they would therefore not reduce current practice workloads, leaving little incentive to support the PCN policy:

> *If it's 'Would you like to use a new workforce to do additional work as well as what you're already doing?", there's no incentive there (SP-1b)*

NHS England's decision to permit a period of prolonged flexibility regarding the utilisation of the new clinical pharmacist workforce, due to the Covid-19 pandemic(9, 31), was widely welcomed. However, there was widespread concern this would give way in favour of a performance management approach in future:

> *Unfortunately, there's a bit of uncertainty about what NHS England are going to want for their money … [attached] metrics don't necessarily align with practice priorities (SP-1)*

Some PCNs decided on operational models that implied least risk for member practices, despite the presence of trade-offs, because of the unclear benefits of the clinical pharmacy workforce. Operational models for employing this new workforce ranged from direct employment by individual GP practices or by a single, typically large general practice holding the employment contracts for all general practices within a PCN (i.e., the 'Lead Practice' model); to indirect employment via a GP Federation (i.e., a pre-existing, formal grouping of GP practices) or contracted from a private provider. While direct employment was widely seen to enhance the capacity of PCNs to manage and direct the new clinical pharmacy workforce, the risk of employment liabilities increased:

For peer review only - http://bmjopen.bmj.com/site/about/guidelines.xhtml

> *There's a lot of anxiety about liabilities for practices because GP partnerships are not limited liability companies … it could be a risk if you end up in an employment tribunal or something like that with risk to the partners … There was a lot of uncertainty about the structure of the networks, how legally it would work, how a contractor would work with each practice and so on, that practices felt a bit uneasy …. To be truthful, we're still negotiating how that's going to work (CD-1)*

PCNs that mapped onto pre-existing GP Federations could initiate processes for recruiting the new clinical pharmacists quickly. This meant they could recruit pharmacists that they considered most suited to the role, in what was widely reported to be a tight labour market. One Clinical Director of a GP Federation-aligned PCN reported, unusually, having to "bat them away" (CD-3a). However, the GP Federation model received contrasting appraisals in the sample, which probably reflects local variation in the capability of these organisational forms to serve as policy vehicles(32). Appraisals included concerns about a lack of operational autonomy and the quality of GP Federation support. One senior pharmacist argued that employment via GP Federation created an "identity problem" for new clinical pharmacists once in post:

> *Unfortunately, our GP Federation isn't a strong one. They don't give any support other than payroll, and they take a management fee… It leaves people with an identity problem. Their employer is the GP Fed but it doesn't do anything other than pay their salary (SP-1b)*

The absence of an optimal operational model meant that PCN decisions about employment arrangements were subject to ongoing debate and experimentation that complicated the early utilisation of the new clinical pharmacists once recruited. While some GPs and practice managers were reported to be skeptical initially, however, positive experiences of the clinical pharmacists, once in post, could increase support for the PCN policy, with subsequent implications for local choices about operational models. One Clinical Director reported that local practices were seeking to pay for additional PCN clinical pharmacists because successful, early integration had given practices more "confidence":

> *It's given practices more confidence to almost try before they buy into it, and take on the employment risks and rights themselves (CD-3b)*

In some PCNs, the new clinical pharmacists' contributions to the Covid-19 response could shift local opinion. One Clinical Director observed an increase in support for direct employment due to the crucial role their clinical pharmacists had served in the primary-care led vaccine programme, which was rolled out during the study period. This had, unexpectedly, presented formative opportunities for their PCN pharmacy team which contrasted with a neighbouring PCN that had decided against direct employment:

> *It became a catalyst, actually, to integrate the team into the PCN. There's nothing else except vaccine, you eat, sleep, dream about vaccine. It would have been impossible without having a pharmacist in the PCN. It made such a difference compared to our neighbouring PCN who hasn't had their own inhouse pharmacist (CD-2b)*

For peer review only - http://bmjopen.bmj.com/site/about/guidelines.xhtml

Theme 2. **Local multi-professional decision-making on clinical pharmacy workforce integration and early task assignment**

A long-term aim of the PCN policy was to implement, at scale, a patient-facing clinical pharmacy role, although there was recognition that the new clinical pharmacy workforce would take time to develop the requisite skills for this. In order to qualify for ARRS funding, newly recruited clinical pharmacists had to undertake formal training for the role, delivered by the Centre for Pharmacy Postgraduate Education (CPPE). This 18-month pathway provided them with an introduction to primary care and training in patient-facing, consultation skills. There was also an expectation that each clinical pharmacist would be assigned a GP supervisor to develop and support them in the role. However, this took place within a context of staff and service pressures and the Covid-19 pandemic. While the primary-care led vaccine programme presented formative opportunities for some PCN pharmacy teams, constraints on in-person interaction were widely held to slow the development of a patient-facing role. One senior pharmacist, who had had 18 years of experience of primary care pharmacy, warned that policymakers' unrealistic expectations were setting up clinical pharmacists to fail:

> *From my understanding of advanced practice, I wouldn't put myself [yet] where they're selling the clinical pharmacists will be in 18-months' time. You're setting them up to fail (SPX-9)*

The senior pharmacists in the sample had roles planning PCN pharmacy services and assisting with recruitment, management and supervision. They highlighted the unique experience, knowledge and skills they brought to this role, including their understanding of primary care pharmacy and, indeed, medications, which GPs do not necessarily have. The supervision they provided was considered complementary to that of GPs as it focused on medication-related questions, with GPs answering diagnostic-related questions. Having gone through a similar process to the new recruits, moreover, they considered themselves well-placed to build confidence and adapt early task assignment to each individual:

> *We decide, almost through shared decision-making with them, what level they're at, sign off their competencies, build it up slowly, give them, not boring tasks but simpler things … And then, from building your confidence, we'll develop you into the clinical pharmacist that … we want in our team (SPX-1)*

Yet, senior pharmacists' autonomy to shape the integration of the clinical pharmacists differed markedly. The extent that Clinical Directors were "pro-pharmacy" or knowledgeable of the clinical pharmacist role was reported to vary:

> *If I compare our PCN against some of the other[s] … within our locality, the Clinical Directors have been pro-pharmacy … The other Clinical Directors have struggled to grasp what a clinical pharmacist can do (SPX-8)*

Many of the senior pharmacists shared concerns about "GP led" PCNs (SPY-3b) which lacked sufficient senior pharmacy input and were purportedly employing clinical pharmacists solely as a means of reducing practice workloads rather than balancing practice priorities with strategic PCN objectives. Although many expressed sympathies

with GPs about longstanding workload pressures in primary care, the senior pharmacists were concerned that, in these PCNs, the initial tasks being assigned were not aligned with the skills, experiences or aspirations of the new clinical pharmacists. Examples of early skill over-utilisation and under-utilisation were reported, with possible implications for staff wellbeing, retention and patient safety. For example, one senior pharmacist described how early, GP-led task assignment may have contributed to medication-related errors:

> *We've just had a massive issue switching everyone from Warfarin to NOACs [novel oral anticoagulants]. The pharmacists didn't really have any training in it, so we've now got people on NOACs that shouldn't be on NOACs. I've found two that need to back on Warfarin. So, they need to have training before they do anything and that seems to be a mismatch (SP-2)*

A more common concern was that new clinical pharmacists were being assigned basic administrative tasks, described as "all the, kind of, mop up jobs for the GP" (SPX-1). The prospect of path dependency in role development was also widely recognised, with some PCNs described as being "stuck in a rut" (SPY-2).

PCN leaderships developed contrasting approaches for integrating clinical pharmacists into general practices, in response to this tension between PCN and practice priorities. Some PCNs opted to have clinical pharmacists float around individual general practices. Under this model, PCN priorities predominated, with the PCN pharmacy team offering set services to practices such that the problem of skill under- or over-utilisation in early task assignment was avoided. Yet, a reported weakness of this model was that the new clinical pharmacists could struggle to form working relationships with GPs and other general practice staff. A different approach was to assign a clinical pharmacist to each individual practice and attempt to balance PCN and practice requirements. This model could result in practice priorities predominating in early task assignment but a reported benefit was that the clinical pharmacists could integrate better into individual general practices and form stronger relationships with general practice staff. As with PCN choice of operational model, approaches for integrating the clinical pharmacists into general practices were subject to change as learning about the roles was acquired locally, and increasing numbers of clinical pharmacists were employed. One senior pharmacist, critical of their PCN leadership's approach, saw an opportunity to rethink this as their Clinical Director stepped down:

> *I think now is the opportunity, with this new Clinical Director coming in, to pull them out, do more training with them and change and direct the role, because they're not practice pharmacists, they're not care home pharmacists, they're not CCG pharmacists: they're PCN pharmacists (SP-2)*

The PCN policy emerging in practice

The findings encapsulate key issues relevant to the early integration of the clinical pharmacist workforce within newly forming PCNs; Figure 1 provides, in diagrammatical form, a cross-theme summary model of policy emergence and adaptation on the ground, based on interviewee accounts.

For peer review only

Figure 1: **Early Implementation of the PCN Clinical Pharmacy Role in Newly Formed PCNs**

[Enter figure]

To the left of the model are those aspects of the national PCN policy that were observed to be stimulating local action and decision-making on the ground, notably PCN funding and the DES contract. The two overlapping circles in the middle represent the core, intertwined processes involved in local implementation: PCN formation and clinical pharmacist integration. The initial plan was for PCNs to form in the first year of the policy and for PCN pharmacy teams to expand thereafter: the overlap, along with the double-ended, curved arrows linking the circles, indicate that PCNs were not developing in such a linear process. Rather, PCNs were still being formed more than 2 years into policy implementation and crucial decisions about operational models extended long into the process of integrating the clinical pharmacists into PCNs. This second process was complicated by the fact that some GPs and practice managers were not sure whether to support it and the absence of an optimal operation model meant that, in some PCNs, clinical pharmacists were being integrated into organisations in a state of flux. Local learning about the possible contribution of clinical pharmacists could, however, lead to decisions pertaining to PCN operational models being revisited, with a view to more optimal integration in future.

The core outcomes, at this stage of the PCN policy, are summarised to the right of model: outcomes were categorised as optimal or suboptimal, based on interviewees' evaluations of clinical pharmacist integration. The double-ended arrows between the outcomes and the circle representing clinical pharmacist integration raise the possibility of feedback loops where an initial success paves the way for further successes as PCN members see the potential of the new workforce. The converse is also illustrated, as early failures are constraining and invite path dependency in role development. Below, the 'moderating factors' are candidate explanatory factors, based on this analysis, for variations in the outcomes across the PCNs in the sample. The external threats identified at the top of the model comprise factors reported to impede progress across PCNs generally, although more advanced PCN pharmacy teams could mitigate their effects or, as in the case of the primary-care led vaccine programme, create opportunities out of them.

Discussion

This paper contributes to scholarship on NHS primary care restructuring and the "mid-level" professional roles that are emerging globally(8, 9). In particular, it highlights the challenges involved in scaling up new roles across a health system, here a primary-care, clinical pharmacy role, being implemented via PCNs, as part of a major policy effort to embed elements of international best practice in the English NHS. The contribution includes examples provided, by senior pharmacists, of clinical pharmacists being over- and under-utilised in their early task assignment, with the allocation of excessive basic administrative tasks being a widespread concern. The complex systems modelling exercise revealed potential for path dependency in role

development, implying a significant challenge to long-term PCN objectives. This is consistent with a recent report from The King's Fund, which highlights considerable variation in emerging PCN practice, amidst a lack of shared, local understanding about the purpose of the new PCN roles(20). This emerging picture of undesirable variation in PCN policy implementation suggests the cogency of concerns voiced earlier on in the policy cycle that policymakers were being overambitious about what could be achieved in a challenging environment(17, 33, 34).

Indeed, Checkland et al's interview study of national policymakers and stakeholders in the PCN policy in its formative stage revealed contrasting policy objectives that, the authors warned, were likely to complicate implementation(17). Notable among these was a tension between the objective of supporting primary care via investment and the objective of facilitating the design and delivery of new services across organisational boundaries. Checkland et al(17) concluded that stronger temporal sequencing of implementation, focusing on the development of primary care first before the delivery of new services, would maximise the likelihood of success. Our findings attest to the clear sightedness of Checkland et al's analysis. We found that local decisions about PCN formation and, in particular, how to employ the clinical pharmacists, were complex and characterised by uncertainties, trade-offs and risk. They extended into the integration of the clinical pharmacists, with potential implications for long-term role development. Somewhat ironically, Covid-19 enforced a stronger version of temporal sequencing, similar to that recommended by Checkland et al(17), as the delivery of PCN services was paused such that a new workforce was available to assist in the primary care led vaccine programme(9, 31). PCNs appear to have had varying degrees of success in integrating the clinical pharmacist workforce under these conditions however. Immediate research and policy implications of the analysis are discussed next, followed by implications for wider debates about NHS governance:

Research implications – The unfolding course of events in restructuring primary care and optimising the contribution of the expanded clinical pharmacist workforce deserve to be studied closely, as do other national policy innovations largely or entirely contingent upon local priorities for delivery. This study's focus on the clinical pharmacist workforce complements emerging research into PCN healthcare role development(20). The key research implications here concern the workforce development needs of clinical pharmacists, how these are met in practice, and with what impacts on the roles that emerge.

Policy implications – The picture painted above of undesirable variation in PCN policy implementation suggests that a comprehensive package of support, as outlined in The King's Fund report(20), is required, if PCNs are to expand the primary care workforce effectively and serve a prominent, coordinating role in the emerging NHS architecture. Support from the new NHS partnership structures known as Integrated Care Systems, also still forming, will be important, including organisational and leadership skills development. An extension of the ARRS funding, beyond the five years planned, would provide struggling PCNs with more time, and may alleviate some of the concern about employment liabilities. This study has also highlighted a particular need for a clearer policy and professional framework for senior PCN pharmacists: their input, though

For peer review only - http://bmjopen.bmj.com/site/about/guidelines.xhtml

variable across PCNs, was shown to be vital to the successful integration of clinical pharmacists and this is likely to continue through role development.

Governance implications – The challenges identified in the study pertaining to PCN formation resonate with the interim findings of a National Institute for Health Research funded study of PCNs which found that their contractual nature presented a major implementation barrier(16). Here, the apparent absence of an optimal PCN operational model and associated local concern about employment liabilities was shown to complicate the early integration of the clinical pharmacists. This would imply that the PCN model is currently failing to realise savings in transaction costs, a key source of efficiency in public administered (as opposed to private or market-based) health systems(35). An alternative, public sector administration approach to primary care development would have been to integrate GP practices within the NHS and to expand the primary care workforce via direct employment by statutory bodies(36). Such an approach may have reduced local exposure to employment liabilities and streamlined the ask of GPs and practice staff to focus on clinical pharmacist integration and role development.

For peer review only

Footnotes

Ethics approval

The study received research ethics approval from NHS Health Research Authority (REC reference 20/HRA/1482).

Funding

This research was funded by the National Institute for Health Research [NIHR] PGfAR [RP-PG-0216-20002]. The views expressed are those of the authors and not necessarily those of the NIHR or the Department of Health and Social Care.

Patient consent for publication

None required

Author contributions

JM, MM and DS designed the study. TM and MM conducted the interviews and analysed the data. All authors made substantial contributions to the theorisation of the data through group discussions and paper development. TM conceptualised and led the write up of the paper; all authors contributed to refining the themes and the model; DS contributed policy expertise; BG contributed methodological expertise.

Competing interests

The Authors declare that there is no conflict of interest

Data availability statement

No data are available. This study has not received ethical approval to share confidential data with any third party other than the study research team.

For peer review only

Bibliography

1. WHO UN. A vision for primary health care in the 21st century [Internet]. World Health Organisation and United Nations Children's Fund. 2018 [cited 2021 Nov 30]. Available from: https://www.who.int/westernpacific/health-topics/primary-health-care

2. Roland M, Guthrie B, Thomé DC. Primary medical care in the United Kingdom. J Am Board Fam Med JABFM. 2012;25 Suppl 1:S6-11.

3. O'Cathain A, Musson G, Munro J. Shifting Services from Secondary to Primary Care: Stakeholders' Views of the Barriers. J Health Serv Res Policy. 1999 Jul 1;4(3):154–60.

4. Doran T, Roland M. Lessons from major initiatives to improve primary care in the United Kingdom. Health Aff Proj Hope. 2010 May;29(5):1023–9.

5. Laurant M, van der Biezen M, Wijers N, Watananirun K, Kontopantelis E, van Vught AJ. Nurses as substitutes for doctors in primary care. Cochrane Database Syst Rev. 2018 Jul 16;7:CD001271.

6. Bradley F, Seston E, Mannall C, Cutts C. Evolution of the general practice pharmacist's role in England: a longitudinal study. Br J Gen Pract J R Coll Gen Pract. 2018 Oct;68(675):e727–34.

7. Drennan VM, Collins L, Allan H, Brimblecombe N, Halter M, Taylor F. Are advanced clinical practice roles in England's National Health Service a remedy for workforce problems? A qualitative study of senior staff perspectives. J Health Serv Res Policy. 2021 Aug 10;13558196211036728.

8. WHO. Global strategy on human resources for health: workforce 2030 [Internet]. World Health Organisation. 2016 [cited 2021 Nov 30]. Available from: https://apps.who.int/iris/bitstream/handle/10665/250368/9789241511131-eng.pdf?sequence=1&isAllowed=y

9. Stewart D, Madden M, Davies P, Whittlesea C, McCambridge J. Structured medication reviews: origins, implementation, evidence, and prospects. Br J Gen Pract. 2021;71(709):340–1.

10. Baird B, Charles A, Honeyman M, Maguire D, Das P. Understanding pressures in general practice. Kings Fund. 2016;100.

11. NHS. NHS long term plan [Internet]. NHS England. 2019 [cited 2021 Nov 30]. Available from: https://www.longtermplan.nhs.uk

12. BMA. The primary care network handbook. British Medical Association; 2020.

For peer review only

13. Leslie M, Khayatzadeh-Mahani A, Birdsell J, Forest PG, Henderson R, Gray RP, et al. An implementation history of primary health care transformation: Alberta's primary care networks and the people, time and culture of change. BMC Fam Pract. 2020 Dec 5;21(1):258.

14. Nuffield Trust. Primary care for the 21st century: learning from New Zealand's independent practitioner associations. Nuffield Trust; 2012.

15. Smith JA, Checkland K, Sidhu M, Hammond J, Parkinson S. Primary care networks: are they fit for the future? Br J Gen Pract. 2021 Feb 26;71(704):106–7.

16. Checkland K, Hammond J, Morciano M, Warwick-Giles L, Lau YS, Bailey S, et al. Primary Care Networks: exploring primary care commissioning, contracting, and provision. PRUComm NIHR Policy Res Unit Health Soc Care Syst Comm. 2021;

17. Checkland K, Hammond J, Warwick-Giles L, Bailey S. Exploring the multiple policy objectives for primary care networks: a qualitative interview study with national policy stakeholders. BMJ Open. 2020 Jul 1;10(7):e038398.

18. Parkinson S, Smith J, Sidhu M. Early development of primary care networks in the NHS in England: a qualitative mixed-methods evaluation. BMJ Open. 2021 Dec 1;11(12):e055199.

19. Warwick-Giles L, Hammond J, Bailey S, Checkland K. Exploring commissioners' understandings of early primary care network development: qualitative interview study. Br J Gen Pract. 2021 Sep 1;71(710):e711–8.

20. Baird B, Lamming L, Bhatt R, Beech J, Dale V. Integrating additional roles into primary care networks. Kings Fund. 2022;

21. Silcock J, Raynor DKT, Petty D. The organisation and development of primary care pharmacy in the United Kingdom. Health Policy Amst Neth. 2004 Feb;67(2):207–14.

22. Guidara A. Complex Systems and Public Policy. In: Guidara A, editor. Policy Decision Modeling with Fuzzy Logic: Theoretical and Computational Aspects [Internet]. Cham: Springer International Publishing; 2021 [cited 2022 Sep 7]. p. 21–34. (Studies in Fuzziness and Soft Computing). Available from: https://doi.org/10.1007/978-3-030-62628-0_3

23. Tian Q. Rural Sustainability: A Complex Systems Approach to Policy Analysis. London: Springer;

24. Mills T, Lawton R, Sheard L. Advancing complexity science in healthcare research: the logic of logic models. BMC Med Res Methodol. 2019 Mar 12;19(1):55.

25. Mills T, Shannon R, O'Hara J, Lawton R, Sheard L. Development of a 'real-world' logic model through testing the feasibility of a complex healthcare intervention: the

For peer review only

challenge of reconciling scalability and context-sensitivity. Evaluation. 2022 Jan 22;13563890211068868.

26. Morris S, Madden M, Gough B, Atkin K, McCambridge J. Missing in action: Insights from an exploratory ethnographic observation study of alcohol in everyday UK community pharmacy practice. Drug Alcohol Rev. 2019 Jul;38(5):561–8.

27. Stewart D, Madden M, Van Dongen A, Watson M, Morris S, Whittlesea C, et al. Process study within a pilot cluster randomised trial in community pharmacy: An exploration of pharmacist readiness for research. Res Soc Adm Pharm RSAP. 2021 Oct;17(10):1750–7.

28. Gale NK, Heath G, Cameron E, Rashid S, Redwood S. Using the framework method for the analysis of qualitative data in multi-disciplinary health research. BMC Med Res Methodol. 2013 Sep 18;13(1):117.

29. Ayres L, Kavanaugh K, Knafl KA. Within-Case and Across-Case Approaches to Qualitative Data Analysis. Qual Health Res. 2003 Jul 1;13(6):871–83.

30. Madden M, Morris S, Ogden M, Lewis D, Stewart D, McCambridge J. Producing co-production: Reflections on the development of a complex intervention. Health Expect. 2020;23(3):659–69.

31. NHS. Structured medication reviews and medicines optimisation: guidance. NHS England. 2020; Available from: https://www.england.nhs.uk/wp-content/uploads/2020/09/SMR-Spec-Guidance-2020-21-FINAL-.pdf

32. McDonald R, Riste L, Bailey S, Bradley F, Hammond J, Spooner S, et al. The impacts of GP federations in England on practices and on health and social care interfaces: four case studies [Internet]. Southampton (UK): NIHR Journals Library; 2020 [cited 2022 Aug 23]. (Health Services and Delivery Research). Available from: http://www.ncbi.nlm.nih.gov/books/NBK554252/

33. Pettigrew L, Mays N. Primary care networks: the risk of “mission creep” calls for focused ambition. BMJ. 2019;367:l5978.

34. Wilson T, Lewis R. Primary care networks: well intentioned but overambitious. BMJ. 2019 Sep 5;366:l5311.

35. Hodgson GM. Towards an alternative economics of health care. Health Econ Policy Law. 2009 Jan;4(Pt 1):99–114.

36. Leys C. The English NHS: from market failure to trust, professionalism and democracy. Soundings. 2017;64:11–40.

For peer review only

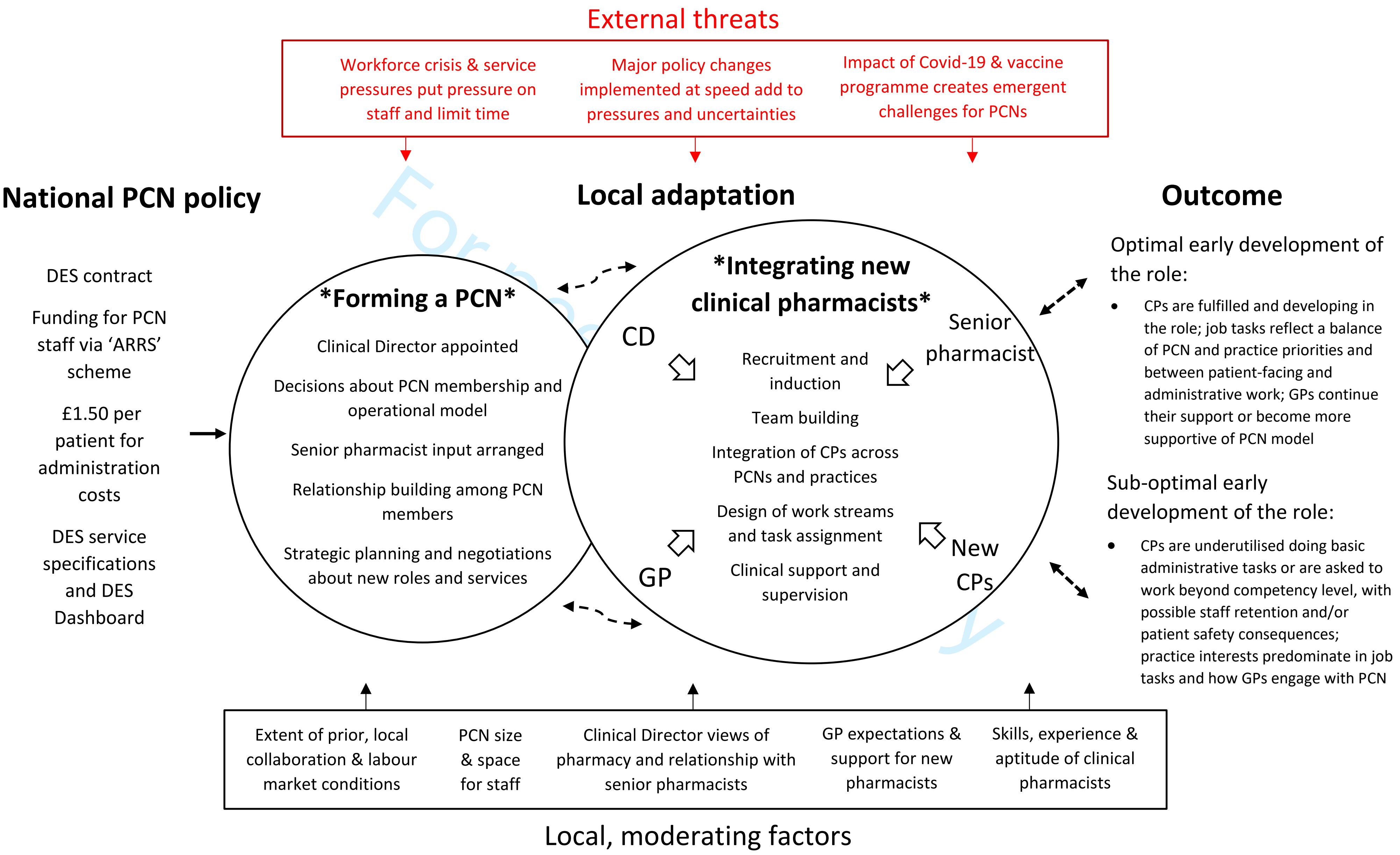
External threats
Workforce crisis & service pressures put pressure on staff and limit time
Major policy changes implemented at speed add to pressures and uncertainties
Impact of Covid-19 & vaccine programme creates emergent challenges for PCNs
National PCN policy
DES contract
Funding for PCN staff via 'ARRS' scheme
£1.50 per patient for administration costs
DES service specifications and DES Dashboard
Local adaptation
Forming a PCN
Clinical Director appointed
Decisions about PCN membership and operational model
Senior pharmacist input arranged
Relationship building among PCN members
Strategic planning and negotiations about new roles and services
Integrating new clinical pharmacists
CD
Senior pharmacist
GP
New CPs
Recruitment and induction
Team building
Integration of CPs across PCNs and practices
Design of work streams and task assignment
Clinical support and supervision
Outcome
Optimal early development of the role:
CPs are fulfilled and developing in the role; job tasks reflect a balance of PCN and practice priorities and between patient-facing and administrative work; GPs continue their support or become more supportive of PCN model
Sub-optimal early development of the role:
CPs are underutilised doing basic administrative tasks or are asked to work beyond competency level, with possible staff retention and/or patient safety consequences; practice interests predominate in job tasks and how GPs engage with PCN
Extent of prior, local collaboration & labour market conditions
PCN size & space for staff
Clinical Director views of pharmacy and relationship with senior pharmacists
GP expectations & support for new pharmacists
Skills, experience & aptitude of clinical pharmacists
Local, moderating factors

Topic guide

- Details of PCN role – job title, when took up post, overview of current roles, brief career history
- PCN policy – views and experiences of PCN policy, relationship with colleagues during transition, autonomy to shape PCN direction, PCN level of maturity, type of operational model, pros and cons of operational model
- New clinical pharmacy workforce – potential of new clinical pharmacy workforce, suitability of national training, personal role in the integration of new clinical pharmacists, local arrangements for integrating, managing and supervising clinical pharmacists
- Medication reviews – personal approach to medication reviews, alcohol within medication reviews, understanding of the new Structured Medication Review (SMR), local implementation of SMRs
- Research – importance of research to PCN leadership, willingness to promote involvement of new clinical pharmacists in a trial of person-centred alcohol discussions in SMRs

For peer review only

Standards for Reporting Qualitative Research (SRQR)*

http://www.equator-network.org/reporting-guidelines/srqr/

Page/line no(s).

Title and abstract

**Title** - Concise description of the nature and topic of the study Identifying the study as qualitative or indicating the approach (e.g., ethnography, grounded theory) or data collection methods (e.g., interview, focus group) is recommended	See page 1
**Abstract** - Summary of key elements of the study using the abstract format of the intended publication; typically includes background, purpose, methods, results, and conclusions	See page 2 – we have followed BMJ Open requirements in writing the abstract

Introduction

**Problem formulation** - Description and significance of the problem/phenomenon studied; review of relevant theory and empirical work; problem statement	We introduce the problem and review relevant research in the introduction – see page 4 and 5
**Purpose or research question** - Purpose of the study and specific objectives or questions	We summarise the research purpose in the final sentences of the introduction – see page 5

Methods

**Qualitative approach and research paradigm** - Qualitative approach (e.g., ethnography, grounded theory, case study, phenomenology, narrative research) and guiding theory if appropriate; identifying the research paradigm (e.g., postpositivist, constructivist/ interpretivist) is also recommended; rationale**	We discuss our complex systems approach on page 5 and include details of our use of the framework method and complex systems modelling – see pages 7 and 8
**Researcher characteristics and reflexivity** - Researchers' characteristics that may influence the research, including personal attributes, qualifications/experience, relationship with participants, assumptions, and/or presuppositions; potential or actual interaction between researchers' characteristics and the research questions, approach, methods, results, and/or transferability	The start of the methods section makes clear that the research was undertaken as part of a broader research programme that is assessing the potential for pharmacists to discuss alcohol within medicine reviews. Links to the research team's publications are provided – see page 5

For peer review only - http://bmjopen.bmj.com/site/about/guidelines.xhtml

**Context** - Setting/site and salient contextual factors; rationale**	We discuss salient contextual factors, notably the challenges of sampling diverse PCNs when they were still forming and changing in composition – see page 6
**Sampling strategy** - How and why research participants, documents, or events were selected; criteria for deciding when no further sampling was necessary (e.g., sampling saturation); rationale**	We describe the sampling strategy through pages 5 and 6
**Ethical issues pertaining to human subjects** - Documentation of approval by an appropriate ethics review board and participant consent, or explanation for lack thereof; other confidentiality and data security issues	We state that all interviewees provided written consent on page 6 and include the REC reference in the footnotes – see page 14
**Data collection methods** - Types of data collected; details of data collection procedures including (as appropriate) start and stop dates of data collection and analysis, iterative process, triangulation of sources/methods, and modification of procedures in response to evolving study findings; rationale**	We describe the data collection methods in detail through the methods section – see pages 6 and 7
**Data collection instruments and technologies** - Description of instruments (e.g., interview guides, questionnaires) and devices (e.g., audio recorders) used for data collection; if/how the instrument(s) changed over the course of the study	We describe the data collection instruments and technologies in detail through the methods section – see page 6 and 7
**Units of study** - Number and relevant characteristics of participants, documents, or events included in the study; level of participation (could be reported in results)	We describe the sample and the level of participation of the participants – see page 6
**Data processing** - Methods for processing data prior to and during analysis, including transcription, data entry, data management and security, verification of data integrity, data coding, and anonymization/de-identification of excerpts	We describe data processes, including an outline of the analytic process from data collection to the analysis stage, with reference to transcription and data coding – see page 7
**Data analysis** - Process by which inferences, themes, etc., were identified and developed, including the researchers involved in data analysis; usually references a specific paradigm or approach; rationale**	The analytic process through which the thematic findings and model were derived is described – see page 6 and 7

For peer review only - http://bmjopen.bmj.com/site/about/guidelines.xhtml

**Techniques to enhance trustworthiness** - Techniques to enhance trustworthiness and credibility of data analysis (e.g., member checking, audit trail, triangulation); rationale**	We outline our approach to ensuring trustworthiness on page 6

Results/findings

**Synthesis and interpretation** - Main findings (e.g., interpretations, inferences, and themes); might include development of a theory or model, or integration with prior research or theory	The main thematic findings are presented first. This is followed by the presentation of the complex systems model which brings together issues and factors described in the themes – see pages 7 to 12
**Links to empirical data** - Evidence (e.g., quotes, field notes, text excerpts, photographs) to substantiate analytic findings	Quotes from the empirical data are threaded through the findings section – see pages 7 to 11

Discussion

**Integration with prior work, implications, transferability, and contribution(s) to the field -** Short summary of main findings; explanation of how findings and conclusions connect to, support, elaborate on, or challenge conclusions of earlier scholarship; discussion of scope of application/generalizability; identification of unique contribution(s) to scholarship in a discipline or field	We draw out the implications of the research in the discussion section and discuss prior research on the topic. We include research and policy implications – see pages 12 to 14
**Limitations** - Trustworthiness and limitations of findings	We discuss the study's strengths and limitations in line with BMJ Open requirements – see page 3

Other

**Conflicts of interest** - Potential sources of influence or perceived influence on study conduct and conclusions; how these were managed	A statement on conflicts of interest is included – see page 14
**Funding** - Sources of funding and other support; role of funders in data collection, interpretation, and reporting	A statement of funding is also included – see page 14

For peer review only - http://bmjopen.bmj.com/site/about/guidelines.xhtml

*The authors created the SRQR by searching the literature to identify guidelines, reporting standards, and critical appraisal criteria for qualitative research; reviewing the reference lists of retrieved sources; and contacting experts to gain feedback. The SRQR aims to improve the transparency of all aspects of qualitative research by providing clear standards for reporting qualitative research.

**The rationale should briefly discuss the justification for choosing that theory, approach, method, or technique rather than other options available, the assumptions and limitations implicit in those choices, and how those choices influence study conclusions and transferability. As appropriate, the rationale for several items might be discussed together.

Reference:

O'Brien BC, Harris IB, Beckman TJ, Reed DA, Cook DA. **Standards for reporting qualitative research: a synthesis of recommendations.** *Academic Medicine*, Vol. 89, No. 9 / Sept 2014
DOI: 10.1097/ACM.0000000000000388

For peer review only

For peer review only - http://bmjopen.bmj.com/site/about/guidelines.xhtml
